# Chronic Pain as an Early Diagnostic Clue in Hereditary Multiple Exostoses: Two Pediatric Cases Highlighting Diagnostic Delay

**DOI:** 10.1002/ccr3.73216

**Published:** 2026-08-13

**Authors:** Melissa Mariti Fraga, Valesca Oliveira Paes Tanaka, Luciana Tudech Salgueiro Pedro Paulo, Maria Carolina Santos, Carolina Costa Figueiredo, Nara Michelle de Araújo Evangelista, Vânia de Fátima Tonetto Fernandes, Guido de Paula Colares Neto

**Affiliations:** ^1^ Darcy Vargas Children's Hospital São Paulo SP Brazil; ^2^ Centro Universitário São Camilo, School of Medicine São Paulo SP Brazil

**Keywords:** chronic pain, hereditary multiple exostoses, osteochondromas, pediatric musculoskeletal disorders, skeletal dysplasia

## Abstract

Persistent musculoskeletal pain in children, particularly when associated with palpable masses or a positive family history, should raise suspicion for hereditary multiple exostoses. Recognizing pain as an early diagnostic clue may reduce delay, enabling timely imaging, structured surveillance, and appropriate long‐term management.

## Abstract (pt)

Hereditary multiple exostoses (HME) is a rare autosomal dominant skeletal disorder characterized by the development of multiple osteochondromas adjacent to the growth plates. Although skeletal deformities and palpable masses are common findings, chronic pain may represent an early and underappreciated diagnostic clue, particularly in pediatric patients. The first patient, an 11‐year‐old boy without a known family history, presented with persistent right foot and knee pain, initially misdiagnosed as a heel spur. Subsequent radiographic evaluation revealed multiple osteochondromas involving the forearms, fingers of the right hand, distal femurs, and bilateral tibias and fibulas. He currently receives symptomatic treatment with analgesics. The second patient, an 8‐year‐old boy, reported chronic lower limb pain since the age of three and had palpable periarticular masses near the joints of the costal grid, fingers, shoulders, forearms, and lower limbs since birth. His mother exhibited similar skeletal abnormalities and short stature. Radiographs confirmed widespread osteochondromas affecting the hands, upper limbs, distal femurs, proximal tibias, fibulas, and pelvis. He remains on daily analgesic therapy without prior surgical intervention. Chronic musculoskeletal pain in children, particularly when persistent or associated with palpable masses or family history, should raise suspicion for HME. Early recognition through careful physical examination and appropriate imaging facilitates timely diagnosis, longitudinal surveillance, and prevention of complications.

## 1 Introduction

Hereditary Multiple Exostoses (HME) is a rare monogenic autosomal dominant disorder with a penetrance of 96 to 100%, typically presenting with a family history, most often from the paternal side. The condition involves the development of multiple benign cartilaginous tumors that arise from the perichondrium, adjacent to cartilage growth. The primary genes implicated are the *Exostosin Glycosyltransferase 1* (*EXT1*) gene, located on chromosome 8, which is detected in 28 to 65% of affected individuals, and the *Exostosin Glycosyltransferase 2* (*EXT2*) gene, located on chromosome 11, found in 21 to 61% of cases [1].

The prevalence of HME is estimated to range from 0.4 to 1 per 50,000 individuals in Western countries, with a predominance in Caucasians. Over 80% of cases are diagnosed before the end of the first decade of life, and previous studies suggest a higher prevalence of HME in males [2].

There is a significant clinical variability due to potential mosaicism of the *EXT1* and *EXT2* genes. As a result, osteochondromas can be associated with reduced skeletal growth, bone deformities, restricted joint mobility, short stature, premature osteoarthritis, and peripheral nerve compression. In some cases, patients may experience chronic pain due to the mass effect, which can also result in sensory or motor deficits [3].

This case report discusses the clinical evolution and management of two patients diagnosed with HME, both of whom presented with chronic pain.

## 2 Case History and Examination

### 2.1 Case 1

#### 2.1.1 Case History and Examination

A Brazilian boy, born to non‐consanguineous parents, was delivered via C‐section at 32 weeks due to maternal hypertension. He required a 32‐day hospitalization post‐birth for low birth weight (1530 g) but did not require subsequent admissions. He was not breastfed, was started on formula feeding after birth, and currently has no dietary restrictions, with regular daily dairy intake.

At the age of nine, he began experiencing symptoms, including pain in the right foot and knee, particularly after physical activity. Initially diagnosed with a heel spur, the persistent pain prompted his mother to seek further medical evaluation. There is no family history of osteochondromas.

At the age of eleven, with continued pain, he presented with an average, proportionate stature (height Z score: −0.08) and an overweight (Body Mass Index Z score: +1.87), with normal growth velocity. Physical examination revealed palpable nodules on his arms, forearms, knees, and ankles. No other clinical or laboratory abnormalities were noted.

#### 2.1.2 Investigation and Diagnosis

**FIGURE 1:**
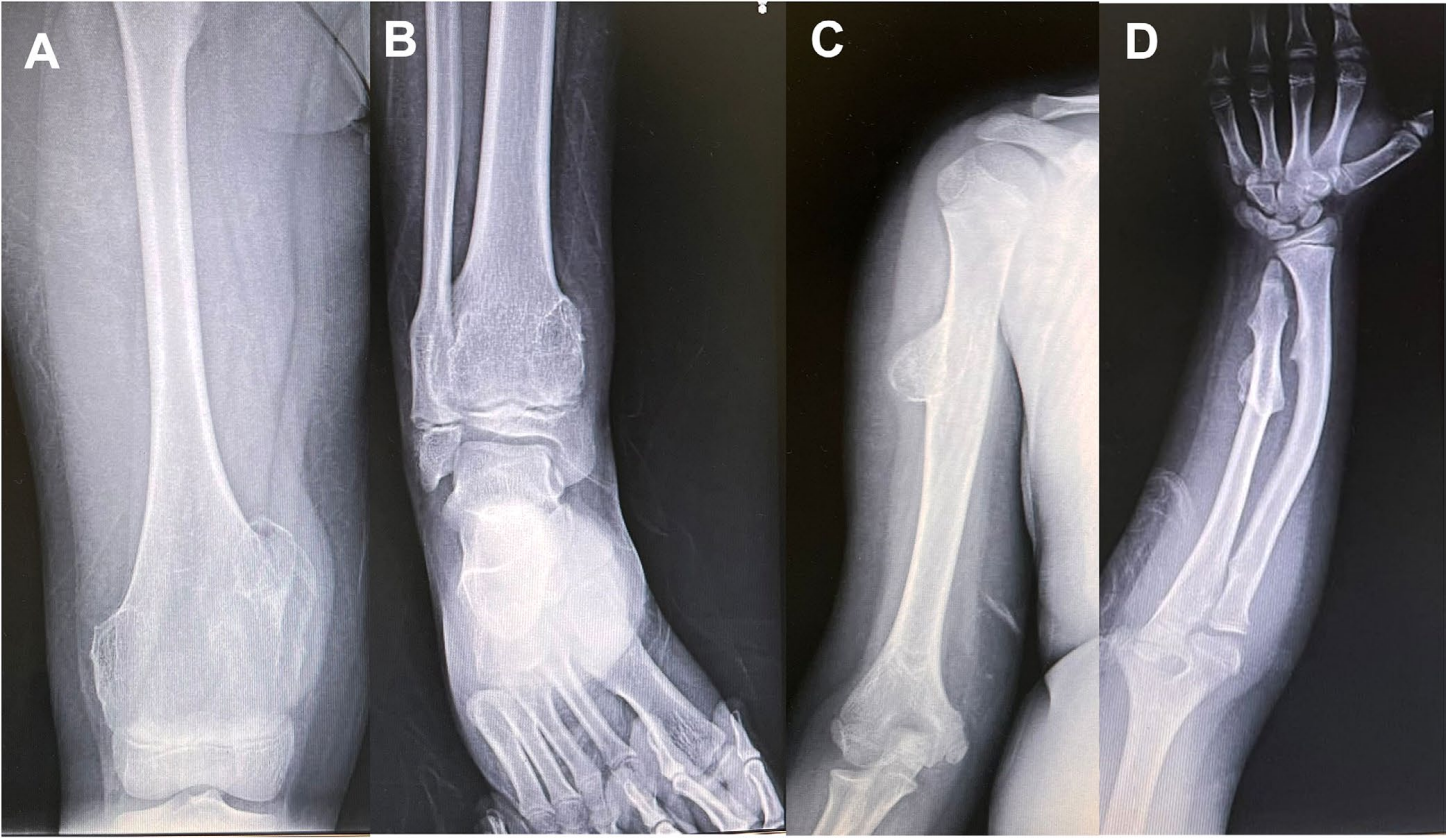
Radiographic images illustrating the presence of osteochondromas. (A) Osteochondroma at the right distal femur. (B) Osteochondroma at the right ankle. (C) Osteochondroma at the right proximal humerus. (D) Osteochondroma at the right distal radius and ulna.

Radiographic evaluation demonstrated multiple osteochondromas, presenting as exophytic bone projections involving the right and left radii, left ulna, right hand phalanges, both humeri, distal femurs, and the proximal and distal tibiae and fibulae. Ulnar asymmetry was noted, with deformity of the distal left ulna (Figure 1). Bone densitometry was not performed. Based on the clinical and radiological findings, a diagnosis of HME was established.

#### 2.1.3 Management and Follow‐Up

He is currently being managed by pediatric endocrinology, rheumatology, and pain management services. He takes daily Gabapentin for pain management and has started swimming lessons. Surgical excision has not been considered at this time.

### 2.2 Case 2

#### 2.2.1 Case History and Examination

A Brazilian boy, born to non‐consanguineous parents, was delivered via C‐section at 39 weeks, with a birth weight of 3500 g, appropriate for gestational age. His mother had no comorbidities or pregnancy‐related complications. He was breastfed until three months of age, after which he was switched to a lactose‐free formula until he was one year old. Currently, his daily intake of milk or dairy products is low. There is no family history of consanguinity, but his mother presents with similar bone deformities and radiological alterations.

Since birth, he has developed palpable nodules, first noted at the costal grid at three months, followed by nodules in his fingers and shoulders at eight months, and in his forearms and lower limbs by the age of three. At three years old, he began experiencing pain in his lower limbs, which worsened with physical activity. By age five, he continued to report chronic pain in his lower limbs, predominantly in the afternoon and at night, though there was no numbness or difficulty walking.

**FIGURE 2:**
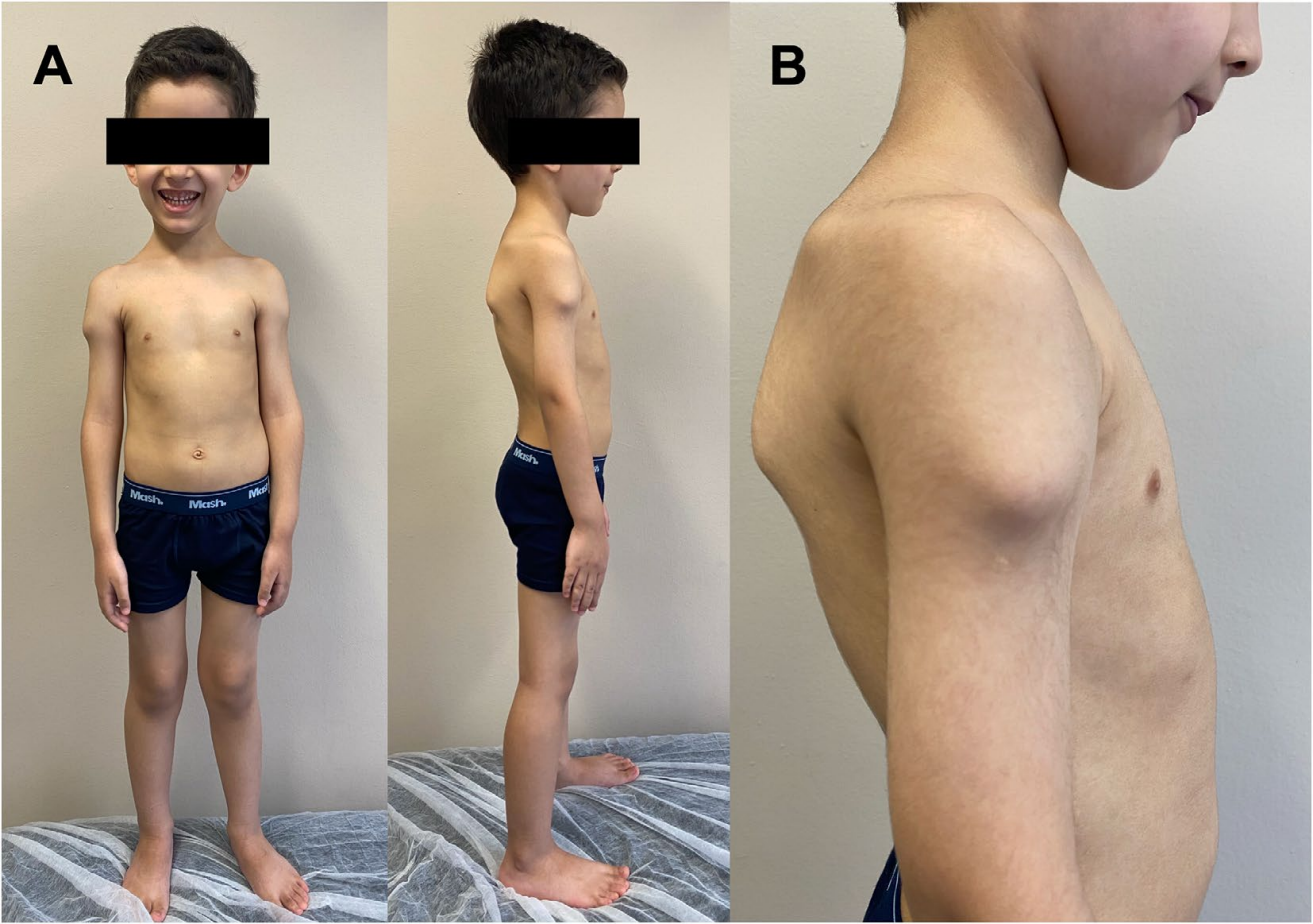
Palpable nodules in the patient at five years of age. (A) Frontal and lateral profile views showing palpable nodules at the wrists and ankles. (B) Close‐up view of palpable nodules in the anterior aspect of the shoulders.

At five years old, he presented with an average, proportionate stature (height Z score: −1.17) and a healthy weight (BMI Z score: −0.72), with normal growth velocity. His physical examination revealed palpable nodules in the wrists and ankles (panel A of Figure 2) and in the anterior shoulders (panel B of Figure 2), which depict the patient in frontal and lateral profile views at five years of age. Additionally, lumbar scoliosis was noted. He exhibited no neurological or dental abnormalities and remained prepubertal. Laboratory tests showed no abnormalities.

#### 2.2.2 Investigation and Diagnosis

**FIGURE 3:**
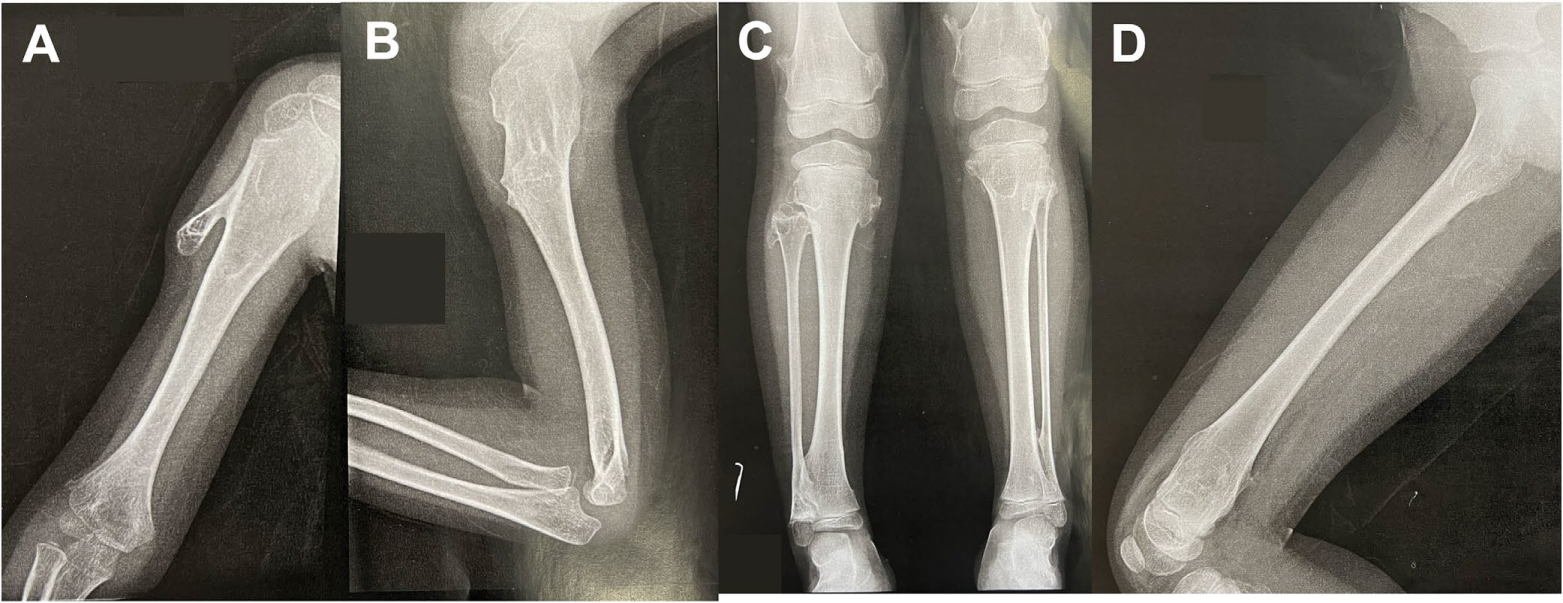
Radiographic images illustrating the presence of osteochondromas. (A) Osteochondromas in the proximal humerus. (B) Osteochondromas in the proximal and distal humerus (lateral view of the elbow). (C) Anteroposterior view of the lower limbs showing osteochondromas in the proximal tibia and fibula bilaterally. (D) Osteochondromas in the distal femur.

Radiographic evaluation demonstrated multiple osteochondromas characterized by large, lobulated, dense osseous masses arising from the metaphyseal regions of long bones. The lesions involved the metacarpals and phalanges of both hands, humeri, radii, ulnae, distal femurs, proximal tibiae, fibulae bilaterally, and the pelvis (Figure 3). No clear medullary continuity was observed on plain radiographs. Bone densitometry revealed average bone mineral density, with a total body–less‐head Z score of −1.1 and a lumbar spine Z score of −1.6. Based on these findings, HME was diagnosed.

#### 2.2.3 Management and Follow‐Up

He is currently under the care of pediatric endocrinology, rheumatology, and pain management services. His treatment includes daily cholecalciferol and gabapentin, along with non‐pharmacological pain relief methods such as warm compresses. He also takes analgesics, such as dipyrone, as needed. To date, he has not undergone surgical excision.

## 3 Discussion

HME is characterized by the development of exostoses or osteochondromas, which are benign bone tumors arising from the abnormal proliferation of chondrocytes in the skeletal growth plates that herniate through the periosteum. These tumors predominantly affect the metaphyses and diaphyses of various bones, including the ribs, scapulae, vertebrae, pelvis, and the long bones of the arms and legs, accounting for up to 50% of benign bone tumors. A cartilaginous cap surrounds these bone tumors, with continuous integration into the original bone's cortex and marrow [4, 5].

The pathogenesis of HME is associated with pathogenic variants in two autosomal dominant genes: *EXT1* on chromosome 8 (locus 8q24.1) and *EXT2* on chromosome 11 (locus 11p11‐13). Although *EXT1* pathogenic variants are globally more common, countries like China and Saudi Arabia exhibit a higher prevalence of *EXT2* pathogenic variants. Approximately 30% of HME cases result from de novo mutations, with the remaining 70% having a familial history. The disease also shows a higher incidence in males compared to females. Among the two patients presented in this report, we observed a male predominance, with one case showing no family history and the other being the son of a mother with the same condition. However, some patients exhibiting the clinical and radiological features of HME may not present with detectable *EXT1* or *EXT2* variants, likely due to mosaic mutations [2, 6].

The abnormal biosynthesis of heparan sulfate, a linear polysaccharide from the glycosaminoglycan family, is the underlying mechanism behind osteochondroma formation. This process, mediated by glycosyltransferases encoded by the *EXT1* and *EXT2* genes, involves the attachment of heparan sulfate to a protein core, forming heparan sulfate proteoglycans. These proteoglycans participate in several cellular processes, including signal transduction, receptor signaling, growth factor activation, cell differentiation, proliferation, angiogenesis, and tumor metastasis [1, 2, 6].

Individuals with HME carry only one functional copy of either *EXT1* or *EXT2*, rendering them susceptible to decreased heparan sulfate synthesis, which leads to osteochondroma formation and abnormal skeletal development. The severity of the disease correlates with the levels of heparan sulfate produced [1, 6].

Exostoses are rarely present at birth, typically developing during childhood and adolescence, with diagnoses most often occurring between the ages of five and seven. On average, patients develop between 15 and 18 osteochondromas, which primarily affect the lower limbs, especially the distal femur, proximal tibia, and proximal fibula. These growths can either be pedunculated, leading to irritation and local trauma, or sessile, attached to the bone by a broader base. The clinical severity of the condition is influenced by the number, location, and morphology of the exostoses. Classification is based on the degree of functional limitation, deformity, and the regions involved (Table 1). In the cases reported here, both patients fall under classification IIB, which aligns with the typical age of diagnosis. Additionally, the number and location of the abnormal bone growths in both cases are within the average range for the condition [6, 7].

**TABLE 1:** Clinical classification of hereditary multiple exostoses [2].

Classification	
I. No deformities and no functional limitations	A. ≤ 5 sites with osteochondromas
	B. > 5 sites with osteochondromas
II. Deformities and no functional limitations	A. ≤ 5 sites with deformities
	B. > 5 sites with deformities
III. Deformities and functional limitations	Functional limitation of 1 site
	B Functional limitation of > 1 site

The initial clinical signs of HME often include difficulties with motor skills, localized pain, and abnormal bone growth. Early presentation of pain, in the absence of visible bone growth, can lead to misdiagnosis, as it is frequently mistaken for growing pains or sprains, particularly when accompanied by a history of pain during movement. Both acute and chronic pain are common complaints in HME, typically resulting from irritation of surrounding structures or joint misalignment. These factors can contribute to degenerative joint disease, often necessitating surgical intervention. Persistent pain—along with diagnoses of chronic inflammation, limited range of motion, or paresthesia and entrapment syndromes—is a frequent issue among HME patients, significantly impacting their quality of life by limiting social interactions, personal activities, and physical exercise [8, 9].

Other clinical features include reduced skeletal growth, short stature, bone deformities, scoliosis, premature osteoarthritis, and peripheral nerve compression. In rare cases, thoracic exostoses may lead to severe complications such as pneumothorax, diaphragmatic rupture, hemothorax, coronary artery compression, and intense chest pain. Additionally, cervical osteochondromas can cause thoracic outlet compression syndrome and dysphagia. Exostoses tend to develop around proximal joints, further restricting movement [2, 5].

In the first case, the patient reported pain in the lower limbs beginning at the age of nine. By contrast, in the second case, palpable nodules were noted since birth, with pain and scoliosis only manifesting at age three. Neither patient experienced complications due to exostoses.

Diagnosis is based on the radiographic and clinical findings of multiple osteochondromas, as well as on the identification of pathogenic variants in the *EXT1* and *EXT2* genes. Imaging studies such as X‐rays, CT scans, and MRI are essential for accurately identifying lesions. In Case 2, no clear medullary continuity was observed on plain radiographs; it is noteworthy that, although classic osteochondromas characteristically demonstrate corticomedullary continuity with the host bone, large lobulated lesions can obscure this feature on plain films. In cases of diagnostic uncertainty or when malignant transformation is suspected, cross‐sectional imaging, particularly MRI or CT, is recommended for definitive confirmation. Although tumor growth ceases in adulthood, periodic MRI follow‐up is recommended given the risk of malignant transformation. Genetic testing, including Sanger sequencing or whole‐exome sequencing, is crucial for genetic counseling. In both reported cases, plain radiographs were critical for establishing the diagnosis following clinical suspicion. However, in the second case, despite the presence of palpable nodules since birth, the diagnosis was only made after complaints of pain and restricted movement prompted further imaging. Genetic testing was not performed in either case due to limitations of the public health system [3, 10].

HME treatment requires a multidisciplinary approach, including family education, discontinuation of harmful medications, promotion of physical activity, and pain management through analgesia. Surgical removal is reserved for symptomatic osteochondromas, with the goals of alleviating pain, preventing deformities, restoring joint function, and addressing circulatory issues. Surgical excision involves removing both the cartilaginous cap and the perichondrium to reduce the risk of recurrence. In cases of severe deformities or limb length discrepancies, more complex surgical procedures may be necessary [3, 6].

In both cases presented, gabapentin was introduced for pain control, with a positive response to treatment. The first patient incorporated physical exercise (swimming lessons) into the treatment plan, whereas the second patient utilized other measures (warm compresses) due to difficulties engaging in physical activities. Surgical intervention was not required for either patient.

For neoplastic lesions, surgical excision remains the primary treatment, as the efficacy of adjuvant radiotherapy and chemotherapy remains inconclusive. Ongoing research is investigating potential treatments, such as palovarotene, a selective agonist that blocks chondrogenesis. Both patients in this report were monitored by a multidisciplinary team and did not present any lesions suggestive of neoplasia [6].

## 4 Conclusion

HME significantly affects patients' quality of life from childhood onward, largely due to chronic pain and progressive skeletal involvement. Persistent musculoskeletal pain, particularly when accompanied by palpable masses or suggestive family history, should prompt early diagnostic investigation. Recognizing pain as a central clinical feature may reduce diagnostic delay and allow timely imaging assessment, structured surveillance, and appropriate multidisciplinary management. Early identification is critical to optimize follow‐up, monitor complications, and improve long‐term outcomes.

## Author Contributions

**Melissa Mariti Fraga:** conceptualization, data curation, formal analysis, investigation, writing – original draft. **Valesca Oliveira Paes Tanaka:** resources, supervision. **Luciana Tudech Salgueiro Pedro Paulo:** resources, supervision. **Maria Carolina Santos:** resources, supervision. **Carolina Costa Figueiredo:** resources, supervision. **Nara Michelle de Araújo Evangelista:** resources, supervision. **Vânia de Fátima Tonetto Fernandes:** resources, supervision. **Guido de Paula Colares Neto:** conceptualization, formal analysis, investigation, writing – review and editing.

## Funding

The authors have nothing to report.

## Consent

Written informed consent for the publication of the submitted article and accompanying images was obtained from the patients and their parents.

## Conflicts of Interest

The authors declare no conflicts of interest.

## Data Availability

Data available on request due to privacy/ethical restrictions.
